# The Death of Dr. Hunter McGuire

**Published:** 1900-10

**Authors:** 


### EDITORIAL NOTES AND COMMENTS.

The Business office of THE JOURNAL-RECORD is No. 64 Marietta Street.

The Editorial office is 318-319-320 The Prudential.

Address all Business communications to Dr. M. B. Hutchins, Mgr.

Make remittances payable to THE ATLANTA JOURNAL-RECORD OF MEDICINE.

On matters pertaining to the Editorial and Original Communications address Dr. Bernard Wolff, Atlanta.

Reprints of original articles will be furnished at cost price. Requests for the same should always be made *on the manuscript*.

We will present, post-paid, on request, to each contributor of an original article, twenty (20) marked copies of THE JOURNAL-RECORD containing such article.

### THE DEATH OF DR. HUNTER McGUIRE.

WE regret to chronicle the death of the distinguished surgeon, Hunter McGuire, of Richmond, Va., which occurred on September 19th. His death was not unexpected, for it was known that the stroke of paralysis he suffered a year ago was the beginning of the inevitable end.

There is perhaps in the medical profession in the South no name more honored or more closely woven with its tragic history than that of Hunter McGuire. It is therefore proper that the notable facts of his distinguished career should be published here, not alone for sectional interest, but that his example may be emulated by younger workers in the field he so greatly enriched.

The sketch of his life which follows is taken from "Physicians and Surgeons of America":

Hunter Holmes McGuire, Richmond, Va., was born in Winchester, Va., October 11, 1835. He was the son of Dr. Hugh Holmes McGuire, and of Anne Eliza Moss, his wife,—the family being directly descended from Thomas Mor McGuire, Lord or Prince of Fermanagh, Ireland, who was born in 1400 and died in 1430.

Dr. McGuire's professional studies were begun in the Winchester Medical College, from which institution he received his degree in 1855. In 1856 he matriculated in both the University of Pennsylvania and Jefferson Medical College of Philadelphia, but was taken sick and compelled to return home before the end of the session. In 1857 he was elected professor of anatomy in the Winchester Medical College, and served one year, and in 1858, feeling the need of greater clinical advantages, he resigned his position in the college, and relinquished a growing practice to return to Philadelphia.

The following year he not only attended the regular course of lectures in the Jefferson Medical College, but also established a quiz class, which was largely patronized by the medical students.

In 1859 the insurrection of John Brown excited the sectional feeling of both North and South, and when his body was taken through Philadelphia, the feeling had become so bitter that a mass-meeting of the Southern students was called, and it was agreed to go to Richmond. Dr. McGuire completed the session in Richmond and in March, 1860, received, for the second time, the degree of Doctor of Medicine. He then went to New Orleans, where he established another quiz class, but after the secession of South Carolina, seeing that the war between the States was inevitable, he hastened home to offer his services to Virginia. He volunteered as a private in Company F, Second Virginia regiment, and April 17, 1861, marched from Winchester to Harper's Ferry. On May 4th he was commissioned as surgeon in the Provisional Army of the Confederate States of America, and assigned to duty as the Medical Director of the Army of the Shenandoah, then under the command of General T. J. Jackson.

General Joseph E. Johnston superseding Jackson in the command of the army, Dr. McGuire served under him until July 1, 1861, when Jackson, having organized the First Virginia Brigade (the future "Stonewall Brigade"), requested that he be assigned to him as brigade surgeon. Dr. McGuire resigned his position under Johnston to accept the one of inferior rank under Jackson, and when the latter was put in command of the Army of the Valley District, he was made its medical director. From this time their relationship was the most intimate; sleeping in the same tent, sharing the same dangers, and undergoing the same hardships—he enjoyed Jackson's entire confidence and esteem. Dr. McGuire was honorably mentioned in every one of the reports of battles made by General Jackson, and one occasion was presented a sword by him, which is now in his possession.

Dr. McGuire soon proved that he possessed the requisite qualifications for his position, for besides his personal skill as an operator, he possessed equally the essential power of organization, and the ability to select competent men to carry out his plans. Never shirking work himself, he demanded the same zeal from his subordinates, and the medical department of Jackson's army soon became famous for its promptness and efficiency.

Dr. McGuire inaugurated the plan of releasing captured medical officers. After the fight at Winchester with Banks, 1862, eight

Federal surgeons were set free upon the simple condition that they would endeavor to procure the release of the same number of Confederate surgeons, and a few weeks after this, all the medical officers who had been confined by both the Confederate and Federal armies as prisoners of war were released and returned to their respective commands. Although this was interrupted by some disagreement between the commissioners for the exchange of prisoners, Dr. McGuire continued to release surgeons whenever it was in his power. Dr. McGuire was also the first to organize the Reserve Corps Hospitals in the Confederate service, and was the originator of the "Ambulance Corps," a system now universally adopted in all armies. In May, 1863, when General Jackson was mortally wounded, Dr. McGuire was relieved from his regular duties as medical director, by order of General Lee, that he might be constantly at the bedside of the dying chieftain. After his death Dr. McGuire served as chief surgeon of the Second Corps of the Army of Northern Virginia, under Lieutenant-General R. S. Ewell, and later on as medical director of the Army of the Valley, under General J. A. Early. He was captured after the battle near Waynesboro, Va., but released under a parole of fifteen days, and after its expiration joined the Second Corps under General J. B. Gordon and remained as its medical director until the surrender at Appomattox. The war being over, Dr. McGuire, in November, 1865, removed to Richmond, having been elected to fill the chair of Surgery in the Medical College of Virginia, made vacant by the death of Dr. Charles Bell Gibson. This position he held until 1878, when the demands of an extensive practice compelled him to resign it; the college conferring upon him in 1880 the title of Emeritus Professor. In 1883, Dr. McGuire established St. Luke's Home for the Sick, a private infirmary for the accommodation of his surgical cases. This institution has grown until now it contains between fifty and sixty beds, and is one of the largest and most successful private sanitariums in the country.

Dr. McGuire's ability as a teacher, his numerous and able contributions to medical literature, his accuracy as a diagnostician and his skill as a surgeon, coupled with his kindness of heart and many noble deeds of charity, have endeared him to the people of his State, and rendered his name a household word in the South. His ability has been recognized both at home and abroad in a most flattering manner, and he has received many honorary degrees and held may positions of eminence. The degree of Doctor of Laws was conferred upon him in 1887 by the University of North Carolina, and in 1888 by the Jefferson Medical College of Philadelphia. He was president of the Richmond Academy of Medicine in 1869; of the Association of the Medical Officers of the Army and Navy of the Confederate States in 1875; of the Virginia Medical Society in 1880; of the American Surgical Association in 1886; of the Southern Surgical and Gynecological Association in 1889, and of the American Medical Association in 1892. He was vice-president of the International Medical Congress in 1876, and of the American Medical Association in 1881. He was associate fellow of the College of Physicians of Philadelphia. He was, also, honorary fellow of the D. Hayes Agnew Medical Society of Philadelphia and of the medical societies of various States, among which may be mentioned Virginia, West Virginia, North Carolina, and Texas.

In 1866, Dr. McGuire married Miss Mary Stuart of Staunton, Va., daughter of the Hon. Alex. H. H. Stuart, who was secretary of the interior under President Filmore, and has a family of nine children.

---

WHY is it that so many medical journals at present are advertising a cement? Is it because they want their subscribers to stick to them, or that they are stuck on the ad.?

